# Poly Cystic Ovarian Syndrome: An Updated Overview

**DOI:** 10.3389/fphys.2016.00124

**Published:** 2016-04-05

**Authors:** Samer El Hayek, Lynn Bitar, Layal H. Hamdar, Fadi G. Mirza, Georges Daoud

**Affiliations:** ^1^Department of Anatomy, Cell Biology and Physiological Sciences, Faculty of Medicine, American University of BeirutBeirut, Lebanon; ^2^Department of Obstetrics and Gynecology, Faculty of Medicine, American University of BeirutBeirut, Lebanon; ^3^Department of Obstetrics and Gynecology, College of Physicians and Surgeons, Columbia UniversityNew York, NY, USA

**Keywords:** PCOS, ovary, insulin, obesity, diabetes

## Abstract

Poly Cystic Ovarian Syndrome (PCOS) is one of the most common metabolic and reproductive disorders among women of reproductive age. Women suffering from PCOS present with a constellation of symptoms associated with menstrual dysfunction and androgen excess, which significantly impacts their quality of life. They may be at increased risk of multiple morbidities, including obesity, insulin resistance, type II diabetes mellitus, cardiovascular disease (CVD), infertility, cancer, and psychological disorders. This review summarizes what the literature has so far provided from guidelines to diagnosis of PCOS. It will also present a general overview about the morbidities associated with this disease, specifically with its more severe classic form. Finally, the review will stress on the various aspects of treatment and screening recommendations currently used in the management of this condition.

## Introduction

Polycystic Ovarian Syndrome (PCOS), also referred to as hyperandrogenic anovulation (HA), or Stein–Leventhal syndrome (Evans and Riley, 1958), is one of the most common endocrine system disorders that affect women in their reproductive age (Azziz et al., 2004). Described since 1935 by Stein and Leventhal (1935), it represents a condition in which an estimate of 10 small cysts of a diameter ranging between 2 and 9 mm develop on one or both ovaries and/or the ovarian volume in at least one ovary exceeds 10 ml (Balen and Rajkowha, 2003).

Systematic screening of women according to the National Institutes of Health (NIH) diagnostic criteria estimated that 4–10% of women of reproductive age suffer from PCOS (Azziz et al., 2004). Although it was previously considered as a disorder of adult women, recent evidence suggests that PCOS is a lifelong syndrome, manifesting since prenatal age. In fact, according to the Rotterdam diagnostic criteria, the prevalence of PCOS in adolescents varies between a minimum of 3% (Hashemipour et al., 2004) and a maximum of 26% (Driscoll, 2003). However, the prevalence of the disease in children is still considered unknown (Kamangar et al., 2015).

The economic burden of PCOS is significantly huge. Around 4 billion dollars are spent annually in the United States to screen for the disease and treat its various morbidities, including hirsutism, infertility, and diabetes mellitus (Azziz et al., 2005). The Australian Health System spends more than 800 million dollars every year to account for the disease (Azziz et al., 2005). Patients with PCOS are twice more likely to be admitted to hospital in comparison to patients without it (Hart and Doherty, 2015). Therefore, accurate and early diagnosis of PCOS is necessary not only to prevent future health comorbidities but also to reduce financial cost and burden (Kamangar et al., 2015).

In this review, we will summarize the most relevant and recent reports related to PCOS, briefly addressing the pathophysiology of the disease, then dwelling in more depth into its diagnostic criteria and their limitations in adolescence. Moreover, we will discuss morbidities associated with the classic form of PCOS and we will provide information about the various treatment regimens and screening recommendations for women living with this condition. Throughout the review, we will emphasize the complexity of PCOS in terms of pathophysiology, diagnosis, morbidities, and the multidisciplinary treatment approach it requires.

## Pathophysiology

### Previous hypotheses

Many hypotheses emerged trying to explain the pathophysiology of PCOS. Initially, excess intrauterine androgen had been thought to be a main culprit in the development of the disease. Yet recently, human studies showed neither an association between excessive prenatal androgen exposure and the development of PCOS in youth (Hickey et al., 2009) nor an elevation in androgen levels in the cord blood of females born to mothers with PCOS (Anderson et al., 2010). Another hypothesis, the adipose tissue expandability hypothesis, suggested that infants with intra-uterine growth restriction (IUGR) and spontaneous catch-up growth might develop decreased tissue expandability, meaning that they cannot store lipids appropriately in their fat tissues. Consequently, insulin resistance might ensue contributing to PCOS and hyperandrogenemia (de Zegher et al., 2009). However, this does not apply for patients with PCOS who did not have IUGR or had it but without spontaneous catch up growth (Ibáñez et al., 1998, 2009).

### A multifaceted disease

The best understanding of the pathophysiology of PCOS deals with it as a multifaceted disease involving uncontrolled ovarian steroidogenesis, aberrant insulin signaling, excessive oxidative stress, and genetic/environmental factors.

An intrinsic defect in theca cells can partially explain the hyperandrogenemia in patients with PCOS. Indeed, women with PCOS have theca cells that, still secrete high levels of androgens due to an intrinsic activation of steroidogenesis even in the absence of trophic factors (Nelson et al., 1999). This intrinsic dysregulation also affects granulosa cells which produce up to 4 times higher levels of anti-mullerian hormone in women with PCOS in comparison to healthy controls (Pellatt et al., 2007; Azziz et al., 2009; Villarroel et al., 2011). Studies also show an elevated number of follicles, primarily pre-antral and small antral follicles, in females with PCOS (Webber et al., 2003). A defect in apoptotic processes in some maturing follicles further increases their count in PCOS patients (Das et al., 2008).

Alternatively, decreased insulin sensitivity attributable to a postreceptor binding defect in the insulin signaling pathways has been identified as an intrinsic component of PCOS, independent of obesity (Dunaif, 1997). It was also reported an alteration in gene expression of some players in insulin signaling pathways by microarray gene analysis (Cortón et al., 2007, 2008). Moreover, PCOS has been associated with increased glycooxidative stress (González et al., 2006) secondary to mitochondrial dysfunction (Victor et al., 2009). Oxidative stress can itself induce insulin resistance and hyperandrogenism in patients with PCOS (Victor et al., 2009).

Familial aggregation of PCOS (Azziz et al., 2004; Chen et al., 2011) and genomic identification of PCOS-susceptibility loci (Chen et al., 2011) support the role of genetics in the etiology of this disease. Some studies showed an inherited component of androgen excess in patients with PCOS (Legro et al., 1998; Escobar-Morreale et al., 2005; Yildiz et al., 2006). Furthermore, a polymorphic marker in fibrillin 3 gene associated with PCOS, D19S884, has been identified in independent sets of families carrying the disease (Urbanek et al., 2007; Segars and Decherney, 2010).

### Evolution

Recently, multiple studies are suggesting that PCOS might start *in utero*, mainly in neonates with risk factors implicated in the development of PCOS. This includes low birth weight (Ibáñez et al., 1998; Melo et al., 2010) and high birth weight infants (Cresswell et al., 1997) who later on catch-up on their growth or constantly increase in weight postnatally (de Zegher and Ibáñez, 2009). Such risk factors, along with a susceptible genetic component, can lead to signs of premature pubarche, premature adrenarche (elevated DHEAS), and metabolic syndrome (insulin resistance and visceral adiposity) (Verkauskiene et al., 2007; Ibáñez et al., 2009). In adolescence, the disease will switch to its more common form with signs and symptoms of hyperandrogenism and/or anovulation. Later on, throughout adulthood, the picture may evolve to any of the various PCOS phenotypes (Rotterdam, 2004). Long term morbidities, including cardiovascular disease (CVD), tend to be more prevalent in the postmenopausal period (Shaw et al., 2008; Wang et al., 2011).

The presentation of PCOS as a model that undergoes a step-wise evolution throughout development puts the complexity of this disease into perspective. Indeed, although science has provided an insight into the origins of PCOS, our understanding of it is still lacking. For instance, the adipose tissue expandability hypothesis cannot account for the disease in infants without IUGR. And since uncontrolled ovarian steroidogenesis that causes hyperandrogenism forms one aspect of the disease, how can we explain the lack of correlation between prenatal androgen exposure and the development of PCOS in humans? Should excessive androgen exposure occur at a susceptible growth window for PCOS to manifest in the future? If this is true, what is the age interval of this susceptibility window? How is the genetic component of the disease inherited and does it exhibit penetrance? Could we possibly, 1 day, screen neonates for PCOS? Supplementary studies are definitely required to shed light over the missing links between the ovarian dysregulation, androgen excess, genetics, and various susceptibility factors that might contribute to PCOS.

## Diagnosis

### Guidelines

**Figure 1:**
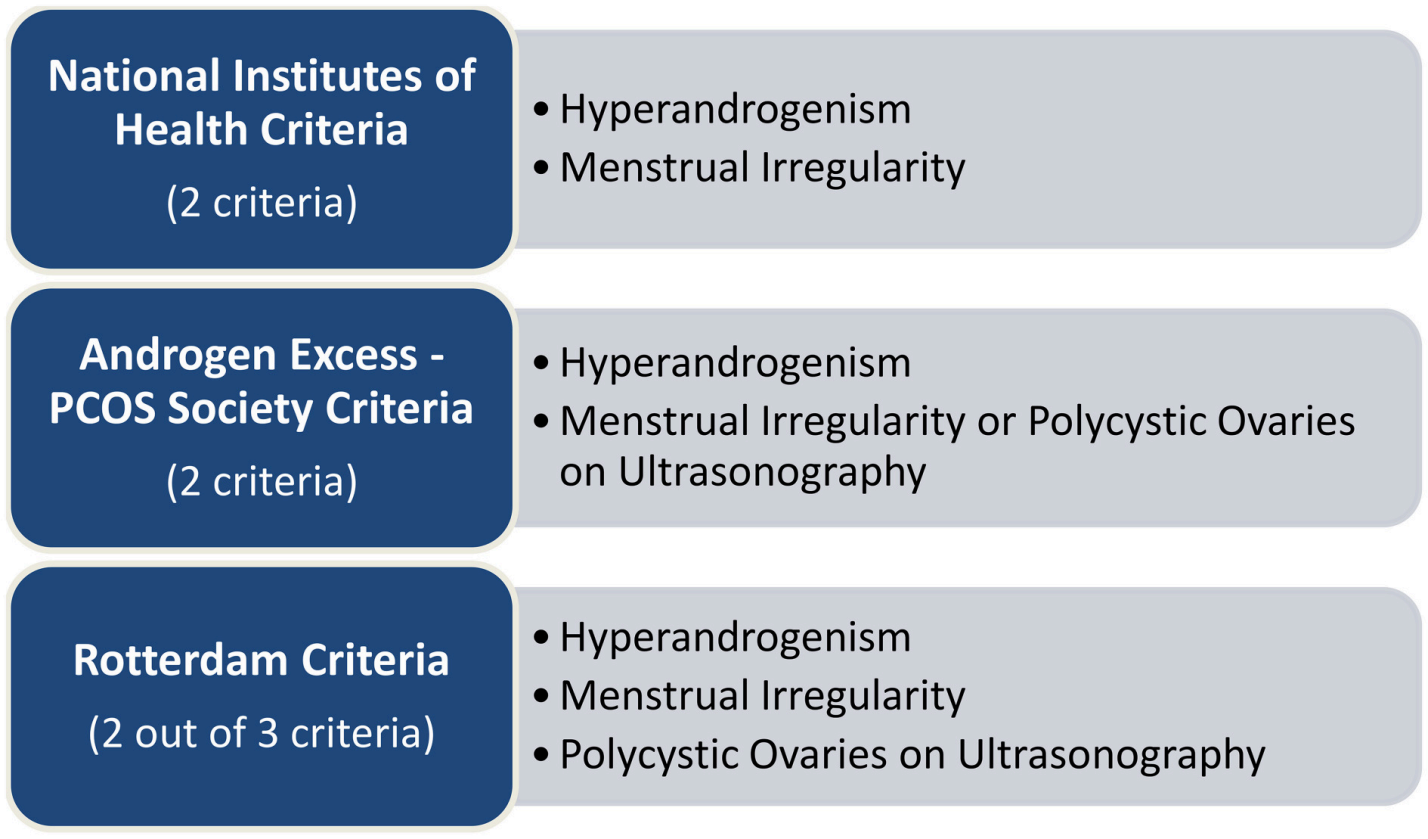
**Guidelines for the diagnosis of PCOS (Rotterdam, 2004; Azziz et al., 2006)**.

Diagnosis of PCOS in adults can follow three different guidelines, which criteria are described in Figure 1. Even though conditions such as insulin resistance and obesity are considered intrinsic to PCOS, none of them is included in the guidelines and should therefore be used for diagnostic purposes (Witchel et al., 2015).

In addition to the information described in Figure 1, each of the guidelines requires ruling out any pathological condition that might explain the hyperandrogenism or the menstrual irregularity (Powers et al., 2015). The disparity between the guidelines, although minor, has been associated with a variation in the diagnosis and the treatment of PCOS (Powers et al., 2015). Moreover, diagnosis in adolescent females is highly debatable (Siklar et al., 2015).

### Signs and symptoms

**Figure 2:**
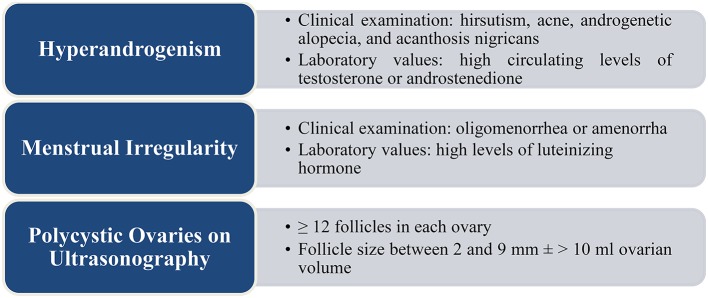
**Signs and symptoms in patients with PCOS (Rotterdam, 2004; Chhabra et al., 2005; Legro et al., 2013)**.

The symptomatic presentation of PCOS usually varies with age, young women mainly complaining of reproductive and psychological problems while older women complaining of metabolic symptoms (Teede et al., 2011). Figure 2 describes signs, symptoms, and laboratory values common in patients with PCOS. A thorough physical examination, medical history, and laboratory tests should be conducted to reach the appropriate diagnosis (Witchel et al., 2015). Discontinuation of spironolactone and oral contraceptive pills (OCP) around 1 month prior to testing, along with testing near the luteal phase of the menstrual cycle are recommended for more accurate results. In addition, on the one hand, testing should include an assessment of the metabolic status of the patient, i.e., measurement of her body mass index (BMI), conduction of a fasting lipid panel, and a 2-h glucose challenge test. On the other hand, screening for thyroid disorders through assessment of thyroid-stimulating hormone levels is considered important as thyroid disorders are a common cause of menstrual irregularity (Kamangar et al., 2015).

### Phenotypes

Since PCOS tends to present as a spectrum of diseases, the Rotterdam criteria divided the disease into four phenotypes (Rotterdam, 2004):

➢ Frank or classic polycystic ovary PCOS (chronic anovulation, hyperandrogenism, and polycystic ovaries)
➢ Classic non-polycystic ovary PCOS (chronic anovulation, hyperandrogenism, and normal ovaries)
➢ Non-classic ovulatory PCOS (regular menstrual cycles, hyperandrogenism, and polycystic ovaries)
➢ Non-classic mild or normoandrogenic PCOS (chronic anovulation, normal androgens, and polycystic ovaries)

Women with the frank phenotype have a worse profile of metabolic and cardiovascular risk factors (i.e., higher insulin resistance and poorer lipid panel) than those with the non-classic phenotype, even when the comparison and the control groups have a comparable BMI (Diamanti-Kandarakis and Panidis, 2007). Likewise, evidence suggests that the frank phenotype may predict a higher rate of postmenopausal cardiovascular morbidity and mortality in comparison to the non-classic phenotype (Shaw et al., 2008). In contrast, women with non-classic normoandrogenic phenotype have less insulin resistant and tend to lack the metabolic features of PCOS in comparison to their counterparts with the classic frank phenotype (Diamanti-Kandarakis and Panidis, 2007; Goverde et al., 2009; Zhang et al., 2009). In this review, the “Associated Morbidities” section will shed light over the complications mostly seen in the classic phenotypes of PCOS rather than in the non-classic ones.

The variation in the symptomatology and presentation of PCOS can account for the presence of different diagnostic guidelines, as the phenotype may vary from being asymptomatic to having signs of all 3 components of the disease (anovulation, hyperandrogenism, and polycystic ovaries). One can argue that the different guidelines might lead to under-diagnosis or over-diagnosis of this condition. This clearly underlines the need for one new single guideline which encompasses all the different phenotypes of PCOS without missing the milder forms of the disease.

## Challenges in diagnosis in adolescents

Held in Amsterdam in 2012, the third PCOS Consensus workshop meeting reported that the diagnosis of PCOS in adolescents should meet the Rotterdam criteria and is therefore similar to the diagnostic features in adults. However, diagnostic practices for adolescents with PCOS tend to be inconsistent and variable among endocrinologists and gynecologists. Diagnosing PCOS in children and adolescence is challenging because the normal pubertal physiological events tend to mimic the signs and symptoms of PCOS (Legro, 2015). This overlap between normal puberty and the diagnostic pathological criteria of PCOS may cause an over-diagnosis of PCOS among adolescent girls (Witchel et al., 2015) which can lead to unnecessary treatment and psychological impairment (Powers et al., 2015).

### Hyperandrogenism

Puberty is characterized by physiological hyperandrogenism (Kahsar-Miller et al., 2001). Multiple studies showed that testosterone levels rise during puberty and reach a peak adult level within a few years after menarche. This can confound with pathological hyperandrogenism and therefore cloud the picture of PCOS (Moll and Rosenfield, 1983; Van Hooff et al., 2000; Mortensen et al., 2009; Rosenfield, 2013). Measurement of testosterone levels does not resolve this uncertainty because testosterone concentrations are highly influenced by the stage of puberty and the menstrual cycle along with other factors (Witchel et al., 2015). In addition, no cutoff values or reference ranges for androgen levels are well defined in female adolescents (Legro et al., 2013). Moreover, acne, which is largely seen during puberty, is not correlated with hyperandrogenism (Hickey et al., 2011). Furthermore, diagnosing hirsutism is challenging since the standardized scoring value does not take into consideration ethnic variations (Ferriman and Gallwey, 1961).

### Menstrual irregularity

Adolescents frequently exhibit physiological menstrual irregularities such as oligomenorrhea (Powers et al., 2015), usually during the first 2 years after menarche, due to lack of maturation of hypothalamic-hypopituitary-ovarian axis (Tfayli and Arslanian, 2008). As such, menstrual irregularity can be sometimes an unreliable criterion for diagnosis of PCOS in adolescents (Kamangar et al., 2015). Through close observation of the menstrual cycle patterns, clinicians have to differentiate physiological anovulation associated with puberty from pathological anovulation as a dysfunction identified in PCOS (Franks, 2002; Wiksten-Almstromer et al., 2008). It has been suggested to postpone diagnosis at least 2 years after menarche to establish a persistent menstrual irregularity (Hardy and Norman, 2013). However, this may delay the initiation of appropriate treatment (Powers et al., 2015).

### Polycystic ovaries on ultrasonography

Normal physiological changes and variations in the volume and size of the ovaries during puberty make ultrasonography findings controversial for the diagnosis of PCOS (Dewailly et al., 2014). Also, performing a transrectal or transvaginal ultrasonography in adolescents may not be always applicable, which may delay diagnosis (Venturoli et al., 1995). For diagnostic purposes, normal ovarian volume in female adolescents is considered equal to or less than 10 ml (Carmina et al., 2010).

## Associated morbidities

### Obesity

Obesity is considered one of the most important features of PCOS. Its prevalence in diseased women varies between 61 and 76% (Glueck et al., 2005). The prevalence of obesity reaches 80% in the United States (Ehrmann et al., 1999) and 50% outside (Balen et al., 1995) which indicates that this figure depends on local environmental factors, ethnic backgrounds, and lifestyle, and not on the mere presence of PCOS itself.

Childhood obesity is a well-documented risk factor for PCOS. Obese girls are at a higher risk of developing insulin resistance, metabolic syndrome, and PCOS later on in life (Pasquali et al., 2011). On the other hand, women with PCOS are at a higher risk of developing obesity (Randeva et al., 2012). Many studies explain that females with PCOS have increased visceral and subcutaneous body fat distribution due to increased androgen production rates (Kirschner et al., 1990); this central obesity follows a masculinized body fat distribution (Borruel et al., 2013) where the amount of visceral fat correlates with the degree of insulin resistance (Karabulut et al., 2012). Moreover, obesity plays a significant role in expressing the metabolic features of PCOS. Women with PCOS have an atherogenic lipid profile, associated with elevated levels of low-density lipoprotein, triglycerides and cholesterol, along with decreased levels of high-density lipoprotein. They are also at a higher risk of developing atherosclerosis, arterial stiffness, and altered vascular endothelium (Hart and Norman, 2006). In addition, women with PCOS show a worsened cardiovascular profile and associated complications (Randeva et al., 2012). However, obesity by itself is not the main reason behind these features. This is evident in lean women with PCOS who demonstrate the same metabolic features as those who are obese (Balen et al., 1995).

Whether obesity leads to PCOS or whether PCOS leads to obesity is still debatable (Kamangar et al., 2015). In animal models of PCOS, administration of testosterone in pregnant rats and monkeys during early conception causes a central abdominal accumulation of fat of the progeny in postpartum life. Hence, prenatal androgen surplus, whether genetic or induced, might be the primordial event in the development of PCOS associated obesity (Escobar-Morreale et al., 2012) yet does not imply that PCOS will ultimately ensue.

### Insulin resistance

A great deal of attention has been given to the metabolic disturbances that accompany PCOS, as well as to the consequences of these disturbances later in life. Today, insulin resistance is considered the main pathogenic factor in the background of increased metabolic disturbances in women with PCOS (Siklar et al., 2015) which can explain hyperandrogenism, menstrual irregularity, and other metabolic manifestations seen in this disease (Baillargeon et al., 2003; Diamanti-Kandarakis and Dunaif, 2012).

In 1980, the association between hyperinsulinemia and PCOS was first noted by Burghen et al. who found a significant positive correlation between insulin, androstenedione and testosterone levels among PCOS women (Burghen et al., 1980). In fact, recent studies show that hyperinsulinemia is present in 85% of patients with PCOS, including 95% of obese and 65% of lean affected women (Teede et al., 2010, 2011; Stepto et al., 2013). Increased insulin levels in patients with PCOS may, along with the high levels of luteinizing hormone, trigger the arrest of follicular growth which contributes to anovulation (Dunaif, 1995). Hyperinsulinemia also alters the gonadotropin-releasing hormone (GnRH) pulse secretion pattern, suppresses the sex hormone-binding globulin (SHBG) and potentiates ovarian androgen production in women with PCOS (Bhattacharya and Jha, 2011; Hart et al., 2011; Huang et al., 2011; Lass et al., 2011; Rathsman et al., 2012; Wedin et al., 2012).

Multiple studies supported a correlation between diabetes and PCOS and showed that insulin-sensitizing drugs and dietary/lifestyle modifications improve hyperandrogenism in patients suffering from PCOS (Baillargeon et al., 2003; Diamanti-Kandarakis and Dunaif, 2012). When the hormone leptin is used as insulin-sensitizing agent, it decreases androgen levels and induces menstruation in affected lean women (Lungu et al., 2012). Other studies showed that 6 months of lifestyle modifications enhanced insulin sensitivity by 70% and significantly reduced anovulation in affected obese women (Baillargeon et al., 2003; Diamanti-Kandarakis and Dunaif, 2012). These studies provide support that insulin resistance aggravates hyperandrogenemia (Lungu et al., 2012). This is one of the critical junctures in the treatment of PCOS, which led to the consideration of insulin-mimetic or insulin-sensitizing agents as part of the management of the disease. These agents, as mentioned later in the review, include metformin, myo-inositol supplements, and thiazolidinedione.

Finally, according to the Diabetes Prevention Program (DPP) Research Group (2002), PCOS patients should be tested for insulin resistance. A few biomarkers have been used to detect insulin resistance in PCOS women. For instance, insulin restrains the release of sex hormone binding globulin (SHBG) from the liver and the production of insulin-like growth factor binding protein 1 (IGFBP-1) (Kalme et al., 2003). It also affects the homeostatic model assessment (HOMA-IR) which is based on computations of fasting glucose and levels of insulin (Legro et al., 2004). Yet, different markers have variable sensitivities and specificities in insulin resistance testing (Nawrocka-Rutkowska et al., 2013). Thus, research should identify one universal marker for accurate diagnosis of decreased insulin sensitivity in patients with PCOS. If such a marker is identified, early detection of insulin resistance can lead to prompt treatment and prevention of any complications in the future.

### Type II diabetes mellitus

PCOS confers a substantially increased risk for type 2 diabetes mellitus and gestational diabetes from early ages (Randeva et al., 2012). About 1 in 5 women with PCOS will develop type II diabetes (Dunaif, 1999) making impaired glucose tolerance a common abnormality in this disease (Randeva et al., 2012). Cross-sectional and prospective longitudinal studies have consistently shown that women suffering from PCOS have a higher risk of developing type II diabetes mellitus or impaired glucose tolerance compared to control populations matched for age and ethnic background (Ehrmann et al., 1999; Legro et al., 1999, 2005; Boudreaux et al., 2006; Talbott et al., 2007; Lerchbaum et al., 2013). Furthermore, prospective longitudinal studies in young and middle-aged women with PCOS show a higher risk for developing diabetes later in life and is mainly due to an increased prevalence of obesity and insulin resistance among these patients (Dunaif, 1999).

Interestingly, family history of diabetes increases the prevalence of type II diabetes mellitus in PCOS patients. However, the prevalence of diabetes in PCOS patients with no family history of diabetes was still much higher than normal women (Ehrmann et al., 1995). Even though family history and obesity are major contributors in the development of diabetes in PCOS patients, diabetes can still occur in lean PCOS patients who have no family history, mainly secondary to insulin resistance (Dunaif, 1999).

### Cardiovascular disease

In 1992, Dahlgren et al. identified a 7 times higher risk of myocardial infarction in patients with PCOS compared to healthy controls (Dahlgren et al., 1992). However, in 1998, an epidemiological study by Pierpoint et al. showed no difference in the risk between the two groups (Pierpoint et al., 1998).

More recent data showed that patients with PCOS have significantly elevated levels of circulating biomarkers of CVD, including C-reactive protein (Bahceci et al., 2004; Meyer et al., 2005) and lipoprotein A (Yilmaz et al., 2005; Bahceci et al., 2007; Berneis et al., 2009; Rizzo et al., 2009), in comparison to matched controls. Other studies demonstrated a higher burden of indicators of atherosclerosis with early onset cardiovascular dysfunction, i.e., arterial stiffness, endothelial dysfunction, and coronary artery calcification (Meyer et al., 2005; Moran et al., 2009).

In 2010, the Androgen Excess-PCOS society provided a consensus statement about increased risk of CVD in women with PCOS and developed a guideline to prevent such complication (Wild et al., 2010). Yet, despite the increased cardiovascular risk markers and the indubitable presence of CVD risk factors in this population, uncertainty remains regarding the increased cardiovascular morbidity and mortality in patients with PCOS (Legro, 2003; Wild et al., 2010; Schmidt et al., 2011; Sathyapalan and Atkin, 2012). Discrepancy between studies might be due to the heterogeneous nature of the populations studied. Therefore, supplementary methodologically rigorous trials are needed to determine the absolute risk of CVD in patients with PCOS throughout age ranges.

### Infertility

Women with PCOS may have reduced fertility (Hart and Norman, 2006; Hart and Doherty, 2015) due to the associated endocrine and gynecologic abnormalities that impact ovarian quality and function (Hart and Norman, 2006). Accounting for up to 90% of ovulatory disorders (Hull, 1987), PCOS-associated persistent periods of anovulation are positively correlated with infertility (Imani et al., 1998). In 1995, a study reported up to 50 and 25% of women in a PCOS population suffering from primary and secondary infertility respectively (Balen et al., 1995). More recently in 2015, a study by Hart and Doherty showed that infertility is 10 times more common among women with PCOS in comparison to healthy controls (Hart and Doherty, 2015).

On the other hand, some studies suggested that females with PCOS who conceive might suffer from pregnancy-related complications such as gestational diabetes (Bruyneel et al., 2014), pregnancy induced hypertension (Hu et al., 2007; Sir-Petermann et al., 2012; Bruyneel et al., 2014), and preeclampsia (Katulski et al., 2015) to a higher extent in comparison to matched controls. Various research data also suggest an increased risk of miscarriage in women with PCOS (Balen et al., 1993; Homburg et al., 1993; Wang et al., 2001; Winter et al., 2002).

The influence of PCOS phenotype, whether classic or non-classic, on female fertility remains poorly comprehended. Data describing the effects of PCOS on pregnancy outcomes are also limited and based on small trials. Thorough studies are needed to assess the degree of infertility in PCOS various phenotypes and to understand the reasons for increased negative pregnancy outcomes in this group of women.

Concerning the effects on the embryo, women with PCOS are 2.5 times at a higher risk of giving birth to small for gestational age children in comparison to healthy females (Katulski et al., 2015) and offspring show an increased morbidity and mortality compared to control (Fauser et al., 2012).

### Cancer

Females suffering from PCOS present many risk factors associated with the development of endometrial cancer, such as obesity, insulin resistance, type II diabetes mellitus, and anovulation (Legro et al., 2013). Anovulation triggers an unopposed uterine estrogen exposure. This can subsequently trigger the development of endometrial hyperplasia and ultimately endometrial cancer (Hart and Norman, 2006). As a matter of fact, studies show that women with PCOS have a three-fold increased risk of developing endometrial cancer (Chittenden et al., 2009; Fauser et al., 2012; Haoula et al., 2012; Dumesic and Lobo, 2013) which is mostly well differentiated with a good prognosis (Fauser et al., 2012). Regardless, no data support ultrasound screening for endometrial thickness in women with PCOS, which comes in agreement with the American Cancer Society against screening for endometrial cancer in patients with average or increased risk. Yet women should be advised to notify their healthcare provider for any spotting or unexpected bleeding (Smith et al., 2001).

On the other hand, there are limited data to support any association between PCOS and breast and ovarian cancer (Chittenden et al., 2009; Fauser et al., 2012).

### Psychological wellbeing

Psychological stress and PCOS have been shown to be intimately related. A vast number of studies showed that women with PCOS are more prone to suffer from psychological disorders such depression (Veltman-Verhulst et al., 2012), anxiety (Jedel et al., 2010; Veltman-Verhulst et al., 2012), recreational drug-related incidents (Hart and Doherty, 2015), disordered eating, and psychosexual dysfunction (Deeks et al., 2010; Teede et al., 2011) in comparison to healthy female controls. In addition, females with PCOS have a lower self-esteem and body satisfaction (Weiner et al., 2004; Himelein and Thatcher, 2006) and subsequently tend to have more psychiatric hospital admissions than controls (Hart and Doherty, 2015). As a result, they display a low quality of life (Jones et al., 2008; Li et al., 2011; Fauser et al., 2012) and are prone to a high degree of emotional distress (Veltman-Verhulst et al., 2012).

It is worth noting that obesity (Elsenbruch et al., 2003; Hahn et al., 2005; Barnard et al., 2007), acne, hirsutism (Weiner et al., 2004; Himelein and Thatcher, 2006) and irregular menstrual cycles (Elsenbruch et al., 2006), all associated with PCOS, are major contributors to the psychological stress that the patients experience due to the challenging of the female identity and her body image (Deeks et al., 2010; Teede et al., 2010, 2011; Dokras et al., 2011; Legro et al., 2013).

Future research should focus the cause-effect relationship between PCOS and psychiatric diseases and more attention should be given to the psychological aspect of this disorder. This is pertinent to clinical care as emotional disturbances can unfavorably affect lifestyle management.

## Treatment

The management of PCOS targets the symptomatology for which patients usually present, anovulation, infertility, hirsutism, or acne being the most common complaints. Treatment usually requires the corroboration of an interdisciplinary team that can include a family practitioner, a gynecologist, and endocrinologist, a dermatologist, a pediatrician, a psychiatrist, and a psychologist.

The treatment section will mainly focus on two major treatment guidelines: the American Task Force (Legro et al., 2013) and the PCOS Australian Alliance Guidelines (Misso et al., 2014).

### Lifestyle changes

Guidelines recommend exercise therapy and calorie-restricted diet as a crucial part of the management of obesity in women with PCOS. In fact, lifestyle modifications are considered as a cost-effective first line treatment and as a necessary adjunct to medication (Legro et al., 2013; Misso et al., 2014).

Excessive weight, as previously mentioned, is associated with adverse metabolic and reproductive health outcomes in women with PCOS. For instance, female fertility significantly decreases with a BMI >30–32 kg/m^2^ (Teede et al., 2011). Multiple small uncontrolled trials have shown that a body weight decrease of as little as 5% regulates the menstrual cycle, improves fertility, reduces insulin and testosterone levels, decreases the degree of acne and hirsutism, and benefits psychological wellbeing (Clark et al., 1998; Knowler et al., 2002; Pasquali et al., 2006; Norman et al., 2007).

However, so far, neither a specific diet nor exercise schedule has been shown to be superior to another in the management of PCOS. In addition, it is difficult to ascertain the effectiveness of such interventions based on the limited data which sometimes address specific subgroups of women with PCOS. Further studies are needed to compare the efficacy of the different lifestyle management techniques (diet alone or exercise alone in comparison to a combination of both) with or without medical therapy for all associated clinical outcomes.

### Medical treatment

If lifestyle changes are not enough to resolve symptomatology, medical treatment is added for better management of the patient's complaints.

#### Oral contraceptive pills

OCP are the most commonly used medications for the long-term treatment of women with PCOS and have been recommended by the Task Force and the Endocrine Society (Legro et al., 2013), the Australian Alliance (Misso et al., 2014), and the PCOS Consensus Group (Fauser et al., 2012) as first-line treatment for hyperandrogenism and menstrual cycle irregularities in women with PCOS.

By suppressing the hypothalamo-pituitary-ovarian axis, OCP decrease LH secretions, increase sex hormone binding globulins, and decrease free testosterone levels (Costello et al., 2007). This addresses hyperandrogenism-mediated symptoms improving acne and hirsutism (Costello et al., 2007), corrects menstrual cycle abnormalities, and provides a mean for effective contraception (Yildiz, 2015). A minimum of 6 months of OCP regimen is usually required to obtain satisfactory results against acne and hirsutism (Yildiz, 2008a). Even though guidelines do not specify the use of one OCP over another (Fauser et al., 2012; Legro et al., 2013), the best choice for symptomatic treatment is considered to be low-dose oral contraceptives that contain anti-androgenic or neutral progestins (Yildiz, 2008a).

A number of clinical trials associated the use of OCP in patients with PCOS with increased risk of insulin resistance (Baillargeon et al., 2003; Legro et al., 2013). Concerns have been also raised about the negative effects of OCP on the cardiovascular profile of females with PCOS (Baillargeon et al., 2005; Lidegaard et al., 2012). Nevertheless, data from randomized control trials and observational studies demonstrated that OCP are indeed effective and safe for the treatment of patients with PCOS (Mendoza et al., 2014) with their benefits outweighing their risks (Yildiz, 2008b).

Both guidelines recommend clomiphene citrate as first line treatment of anovulatory infertility (Legro et al., 2013; Misso et al., 2014). Exogenous gonadotropins, *in vitro* fertilization, and laparoscopic ovarian drilling are considered as second line of management (Spritzer et al., 2015) when clomiphene citrate with or without metformin fail to achieve fertility.

#### Metformin

Metformin (Glucophage), an oral anti-diabetic biguanide drug, acts by impeding hepatic glucose production and increasing the peripheral insulin sensitivity (Bailey and Turner, 1996; Morin-Papunen et al., 1998). The earliest studies on PCOS patients using metformin were performed in 1994 by Velazquez et al. (1994); the results revealed a 35% reduction in the insulin area and a 31% decrease in insulin area to glucose ratio (Velazquez et al., 1994). Some data revealed that metformin does not improve insulin resistance itself, rather it improves glucose effectiveness, i.e., the ability of glucose *per se* to repress endogenous glucose synthesis and stimulate glucose uptake (Pau et al., 2014). Metformin treatment of obese adolescents with PCOS and impaired glucose tolerance proved beneficial in improving glucose tolerance and insulin sensitivity, in lowering insulinemia, and in reducing elevated androgen levels (Arslanian et al., 2002).

In contrast, one study performed by Tang et al. showed no significant change in insulin sensitivity in PCOS patients receiving metformin. This could be explained by the high level of obesity (BMI>30 Kg/m^2^) and the limited weight loss the patients in the study could attain (Tang et al., 2006). Similarly, Ehrmann et al. showed that metformin did not improve insulin resistance in PCOS women (Ehrmann et al., 1997). Acbay et al. stated that metformin has no tangible effect on insulin resistance in PCOS patients (Açbay and Gündoğdu, 1996). Even though studies show contradictory results regarding metformin effect, it is suggested as first-line treatment for cutaneous manifestations and pregnancy complications in women with PCOS. It is also used as a combination with clomiphene citrate to improve fertility outcomes in clomiphene citrate resistant patients (Legro et al., 2013; Misso et al., 2014).

#### Thiazolidinediones

Thiazolidinediones (TZD) represent a class of insulin sensitizer drugs used in the treatment of type II diabetes mellitus. They activate the gamma isoform of the peroxisome proliferator-activated receptor, which is an adipocyte transcription factor (Majuri et al., 2007). The use of pioglitazone (Actos®), one member of this class, was studied in patients with PCOS and data showed that its administration results in a decline in fasting serum insulin levels and insulin resistance (Brettenthaler et al., 2004; Stabile et al., 2014). However, following the association of pioglitazone with increased risk of bladder cancer (Lee and Marcy, 2014; Levin et al., 2015), it has been recommended against its use or the use of other TZDs (specifically troglitazone and rosiglitazone) in the treatment of PCOS due to major safety concerns (Legro et al., 2013).

#### Inositol

Recently, new drugs are being marketed as a novel treatment of PCOS and are gaining more recognition due to their lack of side effects. These are myo-inositol (MYO) and D-chiro-inositol (DCI), 2 stereoisomers of inositol, an insulin-sensitizing molecule.

Growing evidence suggests that insulin resistance might be induced by an alteration of the metabolism of inositol phosphoglycans (IPG) second messengers and mediators or by a defect in their tissue availability (Baillargeon et al., 2008). Many trials demonstrated that MYO administration improves insulin resistance in PCOS patients (Galazis et al., 2011; Morgante et al., 2011). One study reported that the decline in insulin resistance is positively correlated with increasing fasting insulin plasma levels, which supports the role of inositol as a modulator of insulin-mediated metabolic pathway (Genazzani et al., 2012).

More recent studies assessed the effect of MYO in combination with other new drugs. For instance, when combined with monacolin K (natural statin) and lipoic acid, inositol showed a dose-dependent improvement in dyslipidemia and hyperandrogenism-associated symptoms (Morgante et al., 2015). When combined with folic acid, MYO decreased hyperstimulation syndrome to a higher extent than folic acid alone in PCOS females undergoing oocyte retrieval (Papaleo et al., 2009). MYO also improved reproductive outcomes in those undergoing IVF when it was combined with α-lipoic acid (Rago et al., 2015). More importantly, the combination of MYO with DCI in a physiological plasma ratio of 40–1 led to a decrease in the risk of developing metabolic syndrome in obese women with PCOS (Nordio and Proietti, 2012). This has been further reinforced by another study that showed significant improvement in PCOS symptoms, in terms of more menstrual cycle regularity, decreased insulin resistance, better lipid profile, and less acne, upon the use of a MYO-DCI combination (Formuso et al., 2015).

Therefore, a combination of MYO and DCI can be a prospective therapeutic approach for the treatment of women with PCOS. New large trials are needed to assess and compare the effect of MYO and its various combinations to the classic PCOS medications and to check for any undetected long-term side effects.

#### Spironolactone

One study showed that spironolactone, a steroid chemically related to the mineralocorticoid aldosterone, was able to improve insulin sensitivity; it also suggested its use for hyperandrogenism-associated symptoms such as acne and hirsutism (Ganie et al., 2004). However, other studies failed to replicate these results (Dunaif et al., 1990; Ganie et al., 2013). Accordingly, guidelines do not provide any specific recommendations for the use of spironolactone in the management of PCOS; further methodological studies are required to assess any benefit, if existent, for spironolactone in the treatment of this disease.

### Treatment in adolescents

So far, no placebo-controlled randomized controlled trials for the treatment of PCOS in adolescents have been conducted. As such, treatment recommendations mainly represent an extrapolation of adult gathered data and are still highly controversial. Recommendations suggest individualizing treatment of adolescents with PCOS for benefits to outweigh risks. However, these recommendations are not to be applied to girls with precocious puberty due to unestablished risk-benefit ratio in this population (Legro et al., 2013).

The mainstay of therapy for adolescents with PCOS is OCPs, provided as both treatment of hyperandrogenism and as an effective contraception method (Guttmann-Bauman, 2005; Hillard, 2005; Diamanti-Kandarakis, 2010). OCPs normalize menses and decrease acne and hirsutism (Cedars, 2004). Lifestyle therapy and weight loss is also considered as part of the first line treatment, especially in obese adolescents, as it also improves acne and hirsutism. Nevertheless, uncertainty remains regarding the best OCPs and their appropriate duration of use in adolescents (Pfeifer and Dayal, 2003).

Alternatively, metformin has been shown to improve hyperandrogenemia, menstrual irregularity, and insulin resistance in obese and non-obese adolescents with PCOS (Ibáñez et al., 2000; Arslanian et al., 2002; Pfeifer and Dayal, 2003). Yet, the necessary treatment period is still indefinite with conflicting data showing a persistent effect of metformin for only 3 months after discontinuation in one study (Ibáñez et al., 2000) but up to 6 months in another (De Leo et al., 2006).

Finally, treatment of PCOS in adolescents is recommended as OCPs may lower the chance of the patients to develop hyperandrogenism in adulthood (Homburg and Lambalk, 2004) and early lifestyle modifications and metformin therapy have been associated with promising preventative results (Legro et al., 2013).

## Screening recommendations

Appropriate care for the patient requires not only to treat current symptoms but also to prevent any morbidity that might develop later in the future, thus the importance of screening recommendations as an essential part of the management of PCOS. This section will shed light over what is recommended in terms of screening for morbidities in patients with PCOS.

### Screening for type II DM

Women with PCOS should be routinely screened for type II DM. Studies have shown that measurement of fasting blood glucose levels alone under-diagnoses type II DM in patients with PCOS, missing up to 80% of pre-diabetic and 50% of diabetic cases (Salley et al., 2007). As such, guidelines currently recommend screening women with PCOS using an oral glucose tolerance test (Azziz et al., 2006). The screening could be done every 3–5 years (Legro et al., 2013), or every second year in patients with no risk factors for type II DM and annually in patients with risk factors (Misso et al., 2014). Examples of relevant risk factors include age, gender, ethnicity, parental history of diabetes, history of high blood glucose levels, use of antihypertensive medications, smoking, physical inactivity, and waist circumference (Moran et al., 2010).

### Screening for CVD

Women with PCOS should be routinely screened for CVD risk factors. Guidelines recommend cigarette smoking assessment, body weight and BMI measurements to check for obesity, blood pressure monitoring to evaluate for hypertension, and a complete lipid profile panel (total cholesterol, low density lipoprotein cholesterol LDL-C, high density lipoprotein cholesterol HDL-C, and triglycerides levels) to screen for dyslipidemia (Legro et al., 2013; Misso et al., 2014). It is important to note that the Australian guideline dwells in depth in its CVD screening recommendations, indorsing blood pressure measurement annually if BMI ≤ 25 kg/m^2^ or at each visit if BMI ≥ 25 kg/m^2^ and lipid profile assessment every 2 years if initially normal or every year if initially abnormal (Misso et al., 2014).

### Screening for psychological wellbeing

Guidelines recommend screening women with PCOS should be screened for not only depression and anxiety (Legro et al., 2013; Misso et al., 2014) but also for negative body image, eating disorders, and psychosexual dysfunction (Misso et al., 2014).

If screening is positive, the health physician should further assess the problem and refer the patient to a specialist if needed.

## Conclusion

An extended amount of knowledge has been learned about PCOS since it was initially described by Stein and Leventhal (1935). Yet, we are still lacking knowledge about many of its aspects, including its etiology, progression throughout life, spectrum of symptoms, and various morbidities. The pathogenesis of PCOS remains obscure, with unregulated steroidogenesis, insulin resistance, oxidative stress, and genetic factors contributing, possibly from prenatal life, to the disease. Supplementary studies are needed to bridge between the various susceptibility factors that might contribute to PCOS.

The current diagnostic guidelines are still vague and might not detect patients with less severe non-classic phenotypes. The guidelines in adolescents lack specificity, as they might fail to differentiate between normal development and pathogenesis. Since proper diagnosis is a crucial step to initiate treatment and prevent future morbidities, further clinical research should seek not only to update and unify guidelines but also to provide an appropriate rationale for diagnostic tools that can detect all PCOS phenotypes.

Morbidities, more common in the frank PCOS phenotype, emphasize the complexity of this disease as a condition that affects many bodily systems, whether endocrine, gynecological, cardiac, or psychological. Therefore, the management of this varied entity requires a skilled and knowledgeable multidisciplinary team who can achieve best patient outcomes. It is imperative to remember that the treatment of PCOS changes throughout age and should be guided by symptomatology. Early detection of long-term morbidities through appropriate screening tests constitutes an essential part of the management of this condition. Guidelines strongly recommend lifestyle modifications as a critical part of the management. OCPs are the main medication of choice for anovulation and hyperandrogenism; clomiphene citrate is the drug of choice for infertility. Studies assessing inositol stereoisomers' effectiveness should carry on as they may become the new drug of choice for treatment.

In conclusion, we hope this review provided an updated summary that sheds light over the complex nature of PCOS. Future research has to focus on the missing blocks in our growing knowledge about this condition, for that physicians will be able to provide the finest care for patients.

## Author contributions

SE, LB, and LH did the litterature review and participated in the writing of the manuscript. FM and GD participated in the writing, reviewing and finalizing the manuscript.

## Funding

This work was supported by the Medical Practice Plan, American University of Beirut Medical center (MPP-AUBMC).

### Conflict of interest statement

The authors declare that the research was conducted in the absence of any commercial or financial relationships that could be construed as a potential conflict of interest.
